# Capture and X-ray diffraction studies of protein microcrystals in a microfluidic trap array

**DOI:** 10.1107/S1399004715002308

**Published:** 2015-03-27

**Authors:** Artem Y. Lyubimov, Thomas D. Murray, Antoine Koehl, Ismail Emre Araci, Monarin Uervirojnangkoorn, Oliver B. Zeldin, Aina E. Cohen, S. Michael Soltis, Elizabeth L. Baxter, Aaron S. Brewster, Nicholas K. Sauter, Axel T. Brunger, James M. Berger

**Affiliations:** a Department of Molecular and Cellular Physiology, Stanford University, Stanford, CA 94305, USA; b Department of Neurology and Neurological Science, Stanford University, Stanford, CA 94305, USA; c Department of Structural Biology, Stanford University, Stanford, CA 94305, USA; d Department of Photon Science, Stanford University, Stanford, CA 94305, USA; e Howard Hughes Medical Institute, Stanford University, Stanford, CA 94305, USA; f Biophysics Graduate Group, University of California, Berkeley, CA 94720, USA; g Department of Biophysics and Biophysical Chemistry, Johns Hopkins University School of Medicine, Baltimore, MD 21205, USA; h Department of Bioengineering, Stanford University, Stanford, CA 94305, USA; i SLAC National Accelerator Laboratory, Stanford, CA 94305, USA; j Physical Biosciences Division, Lawrence Berkeley National Laboratory, Berkeley, CA 94720, USA

**Keywords:** microfluidics, serial crystallography, XFELs, crystal harvesting, sample delivery

## Abstract

X-ray free-electron lasers (XFELs) promise to enable the collection of interpretable diffraction data from samples that are refractory to data collection at synchrotron sources. At present, however, more efficient sample-delivery methods that minimize the consumption of microcrystalline material are needed to allow the application of XFEL sources to a wide range of challenging structural targets of biological importance. Here, a microfluidic chip is presented in which microcrystals can be captured at fixed, addressable points in a trap array from a small volume (<10 µl) of a pre-existing slurry grown off-chip. The device can be mounted on a standard goniostat for conducting diffraction experiments at room temperature without the need for flash-cooling. Proof-of-principle tests with a model system (hen egg-white lysozyme) demonstrated the high efficiency of the microfluidic approach for crystal harvesting, permitting the collection of sufficient data from only 265 single-crystal still images to permit determination and refinement of the structure of the protein. This work shows that microfluidic capture devices can be readily used to facilitate data collection from protein microcrystals grown in traditional laboratory formats, enabling analysis when cryopreservation is problematic or when only small numbers of crystals are available. Such microfluidic capture devices may also be useful for data collection at synchrotron sources.

## Abstract 2

A microfluidic platform has been developed for the capture and X-ray analysis of protein microcrystals, affording a means to improve the efficiency of XFEL and synchrotron experiments.

## 1. Introduction   

X-ray crystallography is a gold-standard technique for the high-resolution structural examination of biological macromolecules. Crystallographic structure determination suffers, however, from a need for large, well diffracting crystals, a condition that is difficult to satisfy for many samples. Indeed, a common byproduct of crystallization trials is the formation of microcrystals (<15 µm), twinned or clustered crystals or highly radiation-sensitive crystals, all of which pose a challenge to the majority of X-ray diffraction sources. The advent of microfocus sources and cryo-crystallography have helped to provide workarounds to these issues (Hope, 1988 ▶; Smith *et al.*, 2012 ▶). However, neither method can fully address the range of crystal idiosyncrasies that arise in practice. For example, a number of defects, such as impurities, dislocations and mis-alignments of unit cells, can arise during crystal growth. These defects are largely responsible for the partitioning of the crystal into ‘mosaic microdomains’, which in turn can cause greater mosaic spread and poorer quality of diffraction images (McPherson & Kuznetsov, 2014 ▶). As these defects arise with a certain frequency per cubic millimetre of crystal volume, it has been suggested that smaller crystals are more likely to have fewer defects and may thus yield higher-quality diffraction (Cusack *et al.*, 1998 ▶; Kupitz, Grotjohann *et al.*, 2014 ▶), provided that the crystals are large enough to produce sufficient numbers of diffracted photons for a good signal-to-noise ratio of the Bragg reflections. Furthermore, for cryocooled crystals, the process of identifying suitable cryopreservation conditions is laborious, often necessitating the screening of hundreds or thousands of crystals, and sometimes fails. Cryocooling may also restrict macromolecules to lower-energy states (Halle, 2004 ▶; Keedy *et al.*, 2014 ▶; Tilton *et al.*, 1992 ▶), which can mask features evident in structures determined at room temperature (Fraser *et al.*, 2009 ▶, 2011 ▶). For their part, high-intensity micro-focus beamlines can rapidly induce radiation damage, even in cryopreserved samples, sometimes preventing the acquisition of complete data sets from a single crystal.

The advent of X-ray free-electron lasers (XFELs; Emma *et al.*, 2010 ▶) has opened up potentially exciting new possibilities for structural biology by allowing diffraction data collection from protein crystals and microcrystals that were previously unusable (*e.g.* <1–3 µm samples) with conventional synchrotron approaches (Boutet *et al.*, 2012 ▶; Chapman *et al.*, 2011 ▶; Sawaya *et al.*, 2014 ▶). The high brilliance of XFEL beams allows diffraction to be generated in ultrafast (femtosecond-order) pulses, permitting diffraction data to be collected before radiation damage can take place (Chapman *et al.*, 2006 ▶). Studies of largely known systems obtained through XFEL experiments have indicated that the technique has the potential to open new frontiers in structural biology (Boutet *et al.*, 2012 ▶; Liu *et al.*, 2013 ▶; Redecke *et al.*, 2013 ▶; Kupitz, Basu *et al.*, 2014 ▶; Sawaya *et al.*, 2014 ▶; Chapman *et al.*, 2011 ▶; Kern *et al.*, 2012 ▶, 2013 ▶, 2014 ▶).

**Figure 1:**
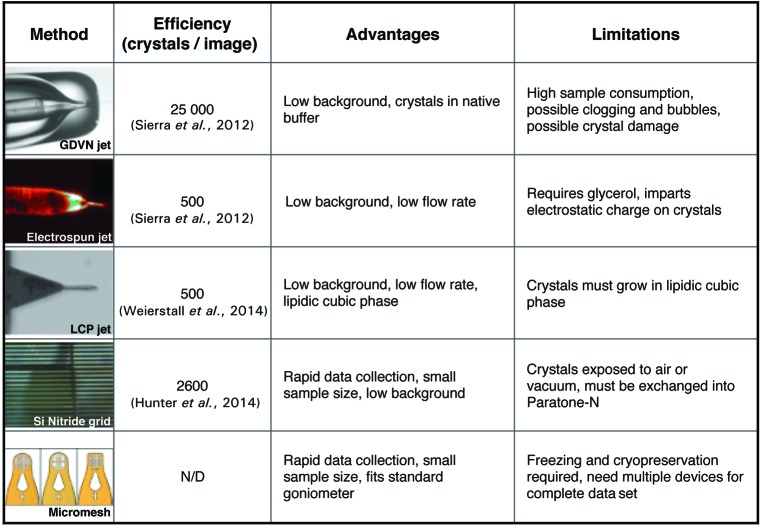
Sample-delivery methods. A comparative summary is shown for various existing liquid-jet and fixed-target sample-delivery methods used with serial femtosecond crystallography at XFEL lightsources. Crystals per image efficiencies were estimated based on the information provided in the references indicated.

Current XFEL-based data-collection strategies face several challenges. One is sample delivery (Fig. 1 ▶). One major means of delivering samples to XFEL sources is a liquid jet (Weierstall, 2014 ▶), in which a stream of crystals (suspended in a slurry) are pushed at high velocity through a rapidly pulsing (currently up to 120 Hz at the LCLS) XFEL beam (DePonte *et al.*, 2008 ▶; Sierra *et al.*, 2012 ▶; Weierstall *et al.*, 2014 ▶). The technique, which is conducted in a vacuum, has the advantage of giving rise to very low background scattering, since each crystal is surrounded by only a thin layer of buffer solution. The efficiency of the method, however, is relatively low: since crystals cannot be individually targeted in mid-flight, the likelihood of the beam intersecting a single crystal and yielding a useful diffraction pattern (termed the ‘hit rate’) rarely exceeds 5%, resulting in the loss of a large proportion of sample (>99%) to waste. Liquid-jet experiments also typically require large amounts of microcrystalline slurry (up to the millilitre range; Chapman *et al.*, 2011 ▶; Sierra *et al.*, 2012 ▶; Weierstall *et al.*, 2014 ▶). This level of sample consumption places the use of liquid jets outside the range of the majority of projects. Finally, liquid jets can suffer from clogging of the stream nozzle with crystals, and fragile crystals may be damaged by the pressures and shear forces inherent to the injection process itself (Stevenson *et al.*, 2014 ▶). Some of the difficulties inherent to liquid jets are being addressed by the development of ‘drop-on-demand’ methods and lipidic cubic phase injectors (Weierstall, 2014 ▶), but lowering the sample consumption into the microlitre regime, working with viscous solutions or mixtures of crystals of varied shapes and sizes, and synchronizing droplet ejection with XFEL pulses all remain persistent obstacles.

An alternative sample-delivery approach to liquid-jet delivery is a goniometer-based, fixed-target system (Cohen *et al.*, 2014 ▶; Hirata *et al.*, 2014 ▶). In this method, a standard goniometer setup is used in conjunction with crystal-coated, cryopreserved micromeshes. The approach allows automated sample mounting from cold-storage conditions and further permits rapid screening and the relatively efficient collection of data with much reduced sample (Cohen *et al.*, 2014 ▶; Fig. 1 ▶). The method is also versatile, permitting all samples (including larger crystals captured in cryoloops) to be attached to a standard goniostat-compatible magnetic base with little or no customization required. Recent experiments with a variety of crystals indicate that goniometer-based sample delivery is a robust and promising approach (Cohen *et al.*, 2014 ▶; Hirata *et al.*, 2014 ▶); however, the use of micromeshes also can present a problem, as it is necessary to cool the sample prior to mounting to prevent dehydration during data collection. This requirement necessitates screening for optimal cryopreservation conditions, thereby obviating one of the major advantages of the ‘diffraction-before-destruction’ approaches that XFELs afford. An alternative fixed-target method, utilizing grids etched into silicone nitride-coated wafers, has recently been demonstrated to enable rapid data acquisition and low sample consumption while making use of crystals that did not have to be cryopreserved (Hunter *et al.*, 2014 ▶). However, the crystals had to be transferred into Paratone-N to protect them from dehydration, a treatment that not all crystals can tolerate.

To promote rapid and efficient data collection from XFEL sources, an ideal sample-delivery method would minimize sample consumption, maximize the crystal hit rate and avoid cooling or other preservation treatments altogether. To begin to address these issues, we have developed a microfluidic device that can be used to capture individual protein microcrystals injected from a crystal slurry in regularly spaced traps. The entire chip can be mounted in front of an X-ray beam and allows high-resolution diffraction data to be collected at room temperature from crystals immobilized in well defined, ‘addressable’ positions within the trap array. We have used this device to extract and array single hen egg-white lysozyme microcrystals from a slurry and to collect high-resolution diffraction data from the captured crystals using both a synchrotron and a XFEL light source. Importantly, the chips use less than 5 µl sample per chip and can work with crystalline solutions grown using any standard laboratory approach, such as hanging or sitting drops or batch crystallization. We demonstrate that diffraction data collected serially from hundreds of single crystals at room temperature under pseudo-XFEL conditions (*i.e.* using stills rather than oscillations) are of sufficient quality to enable the determination of a protein structure. We expect that our microfluidic capture design may have utility for data collection at both XFEL and synchrotron sources.

## 2. Materials and methods   

### 2.1. Chip fabrication   

To fabricate chip molds, we patterned a 10 µm high SU-8 photoresist (Microchem) layer onto a 4′′ silicon wafer (Silicon Quest International Inc.) by standard photolithography techniques using a laser-etched chrome mask (Front Range PhotoMask). After hard baking and silanization of the mold surface, molds were spin-coated with poly(dimethylsiloxane) [PDMS; RTV 615 (part A:B = 20:1), R. S. Hughes] at 2000 rev min^−1^ for 30 s, resulting in a 50 µm thick PDMS layer. After 5 min of degassing and 10 min of planarization steps, the wafers were partially cured for 40 min at 80°C. Concurrent with the preparation of the patterned molds, 2 mm thick blank PDMS layers [RTV 615 (part A:B = 5:1)] were cast by pouring PDMS onto a blank silicon wafer and partially curing for 40 min at 80°C.

Following fabrication, an ∼5 × 15 mm aperture was next cut into each chip to serve as an X-ray window. The window layer was aligned onto the thin layer and the two layers were bonded for 80 min. The bonded layers were carefully peeled off from the wafer and diced into individual 10 × 25 mm sections with a guillotine blade; 20-gauge ports were subsequently punched at inlet and outlet positions. The 2 mm thick PDMS layer enabled easy handling of the chip during hole-punching, bonding to the substrate and crystal loading. Owing to its low background X-ray diffraction, a 250 µm sheet of poly(methyl methacrylate) (PMMA; Goodfellow, Coraopolis, Pennsylvania, USA) was used as a backing substrate. To facilitate bonding and prevent delamination during loading, the PMMA substrate was coated with a 50 µm layer of PDMS. This arrangement results in the flow channel being embedded in a monolithic PDMS–PDMS interface, which obviates interlayer adhesion problems (Unger *et al.*, 2000 ▶) and allows the chips to withstand the back-pressure that would build up during loading. Assembled chips were cured overnight at 80°C. The PMMA substrate was then trimmed to line up with the PDMS layers and the chip was affixed to a standard magnetic base typically used for cryo-loops (Hampton Research). To accommodate the chip, a 2.5 mm slot was machined in the base such that the flow channel is centered with respect to the base.

### 2.2. Crystal growth and trapping   

Hen egg-white lysozyme (HEWL) microcrystals were generated as described by Falkner *et al.* (2005 ▶) except that (i) a highly pure, commercially available preparation of HEWL was used as sample material (EMD Millipore, 5950-5GM) and (ii) the cross-linking step was omitted to avoid damaging the crystals. Lyophilized lysozyme was resuspended at 20 mg ml^−1^ in sodium acetate pH 3.5, split into 50 ml aliquots and stored at −20°C. For crystallization, the lysozyme solution was thawed and spun for 5 min at 13 000 rev min^−1^ (Eppendorf, 5415D) to remove any precipitate or fine-particulate matter. The clarified solution was then transferred to a fresh microcentrifuge tube and crystallization solution (20% sodium chloride, 8% polyethylene glycol 8000, 0.5 *M* sodium acetate pH 3.5) was added at a ratio of 1:3 protein:buffer. Crystals of ∼10–15 µm in size typically formed within 10 min. Smaller crystals could be obtained by chilling the crystallization buffer to 4°C and incubating the batch at the same temperature, whereas larger crystals could be obtained at room temperature. Crystal size could be further fine-tuned by mixing and matching the various temperature treatments.

Crystals were loaded into microfluidic chips using a syringe pump (Harvard Apparatus). To determine the loading efficiency, a simplified version of the chip was used which lacked the X-ray window and was bonded to a PDMS-covered glass slide to permit inspection on a microscope stage (Olympus CKX41). For diffraction experiments, loading was monitored using a variety of microscopes available at the beamlines. In all cases, chips were first primed by injecting crystallization buffer from the outlet towards the inlet using a 25 µl threaded-plunger syringe (Hamilton 1702LT), which was connected to the outlet by a short length of tubing (Weico Wire ETT-26) that inserted directly into the outlet opening. The tubing outer diameter was matched to the gauge of the inlet and outlet openings, creating a watertight seal. Buffer was slowly injected by hand until all of the channels were filled, all bubbles were pushed out of the flow cell and a meniscus formed at the inlet. The syringe was then disconnected; the tubing was left in the outlet and directed towards the outflow collection tube.

After chip priming, the crystal slurry was drawn into another length of tubing pre-filled with crystallization buffer using a 50 µl plunger-operated syringe (Hamilton 1705TLL). Since the crystals tend to settle, the slurry had to be agitated by repeated cycles of gentle plunging and ejection. The syringe was then placed into the syringe pump, the tubing inserted into the inlet and slurry pumped through the chip at a flow rate of 0.5 µl min^−1^. This flow rate was established as optimal for lysozyme microcrystals, which permitted flow through the channels without overly rapid insertion of crystals into the traps. While this procedure worked well with several types of non-lysozyme crystals, we expect that the flow rate will likely need to be adjusted empirically when working with particularly heavy or fragile crystals or with viscous buffers.

Loading experiments were monitored and video-recorded for future analysis. After loading was complete, the chip was further photographed for visual inspection of trapped crystals. For X-ray diffraction studies, the inlet and outlet tubing was cut close to the chip and sealed with epoxy; the chip was then affixed to the magnetic base as described above and mounted on the goniometer in preparation for data collection.

### 2.3. Estimation of trap efficiency   

Assuming that all crystals flowing through the trap array can be captured at a trap site, the capturing efficiency of each individual trap can be calculated as the probability of the crystal taking the trap channel *versus* the bypass channel (Chung *et al.*, 2011 ▶). This probability can be expressed in terms of a volumetric flow ratio,
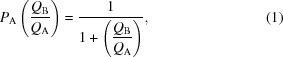
where *Q*_A_ is the volumetric flow rate through the trap channel and *Q*_B_ is the volumetric flow rate through the bypass channel. If we approximate the same pressure differences across the trap channel and the bypass channel, we can express the volumetric flow ratio (*Q*_B_/*Q*_A_) as
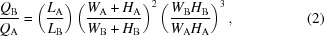
where *L* is the channel length, *W* is the channel width and *H* is the channel height. These equations assume a random distribution of crystals throughout the flow channel. It should be noted, however, that in our trap-array design crystals are ‘guided’ towards the trap using a relatively narrow, slanting channel that approaches the trap at a 30° angle. This feature may help ensure the observed high rate of crystal capture.

### 2.4. Diffraction data collection and crystal structure determination   

Diffraction data were collected by X-ray exposure of trapped crystals in the chip. XFEL diffraction images were collected at the Linac Coherent Light Source (LCLS) X-ray Pump Probe (XPP) endstation. Since XFEL time was limited to a single brief testing period (∼2 h), only a few test images were obtained at XPP using a 80 fs pulse with 3 µm beam size. A Rayonix MX170-HS detector was used to record XFEL images. Microfocus synchrotron images were collected on beamline 12-2 at Stanford Synchrotron Radiation Laboratory (SSRL). A 10 × 10 µm beam size was utilized for all experiments, using 5 s exposures yielding 2.0 × 10^12^ photons s^−1^. *RADDOSE*-3*D* (Zeldin *et al.*, 2013 ▶) was used to estimate an average total X-ray dose of 2.4 MGy for a 10 × 10 × 15 µm crystal. A Dectris PILATUS 6M detector was used to record the diffraction images.

Data used for structure solution were obtained entirely on SSRL beamline 12-2 from 16 chips. Since automated data-collection software for the crystal-capturing chips is currently in development, each trapped crystal had to be targeted manually, which limited the number of images that could be collected during the 24 beamline hours allocated to this work. The trap arrays are much larger than a typical cryo-loop, for which the beamline goniometer was optimized, and hence required more time for translation and targeting. As a result, no more than 10–20 diffraction images could be collected before the flow channel dehydrated owing to water diffusion into the PDMS material. The dehydration caused a rapid flow of bubbles that cleared the channels and traps of crystals and forced us to cease data collection and mount a new chip. These problems, which contributed to limiting the completeness of the collected data, can be addressed in the future by adjustments to the beamline hardware, the development of automated data-collection software and improvements to sample-loading techniques, including pre-hydrating the PDMS chips to reduce or eliminate dehydration. Fortunately, the chips appeared (by visual inspection) to be mounted solidly in place, and did not sag, bend or detach. Furthermore, no shifting of trapped crystals was observed during chip handling or mounting. Once the chip was sealed, all movement in the channels ceased, presenting the crystals as stationary targets. While we did not observe any slippage by eye, we cannot rule out slippage on the submillimetre level. We are designing a new chip holder with this possibility in mind and are exploring goniometer settings that would address any persistent slippage problems.

To randomize the orientation of trapped crystals *versus* the incident beam, chips were rotated 0, 5, 10, 15, 25 and 30° in both clockwise and counterclockwise directions, yielding a total rotational range of up to 60°. Images were indexed and integrated using the *cctbx.xfel* software suite (Hattne *et al.*, 2014 ▶). Of the 265 collected images, 232 could be integrated to variable resolution limits (Supplementary Fig. S1 and Supplementary Table S1). Of these, 212 images were successfully scaled, merged and post-refined using *PRIME* (Uervirojnangkoorn *et al.*, 2015 ▶), yielding an 83.0% complete data set to 2.5 Å resolution. While the resolution cutoff for conventional oscillation data sets is conservatively placed where CC_1/2_ ≃ 0.3–0.5, the variable nature of the serial diffraction images may affect the sensitivity and usefulness of the CC_1/2_ statistic. Consequently, we empirically selected a resolution where refinement was the most stable, with overall completeness >80% and an average of two observations per Miller index. CC_1/2_ decreased relatively smoothly up to this resolution and became erratic in higher resolution bins. Much of this behavior can be attributed to the missing cone of diffraction data as well as the particularly small number of reflections yielded by the tetragonal HEWL crystals. Furthermore, as *PRIME* is still under development and this is the first time, to our knowledge, that *cctbx.xfel* has been used to process serial ‘pseudo-still’ images (*i.e.* 0.02° oscillation) collected at a synchrotron source rather than at an XFEL source, there are a number of beamline-specific parameters (such as beam energy and polarization) that must be taken into account for optimal data processing. Efforts are under way to modify *PRIME* to take these parameters into account.

Although the unit-cell parameters for tetragonal HEWL are well documented in the literature and the Protein Data Bank, we felt it necessary to simulate an unknown unit cell to determine whether the data set obtained from the chips would be sufficiently robust for structure solution. To this end, we carried out the first round of indexing and integration without supplying any target unit-cell parameters. The point group *P*4, as well as the typical unit-cell parameters of tetragonal HEWL, were immediately identified by *cctbx.xfel* for at least a third of the images. We then used these parameters as targets for a second round of indexing and integration. However, *PRIME* does not yet include a systematic absence-analysis method, and moreover scaling statistics alone may not unambiguously determine the space group of the merged data set. To determine the space group, we therefore performed two separate rounds of molecular replacement (MR) with *Phaser* (McCoy *et al.*, 2007 ▶) using a room-temperature structure of lysozyme (Pinker *et al.*, 2013 ▶) from which all water molecules, heteroatoms and side chains had been removed. In each case, *Phaser* was instructed to search for solutions in all space groups within each of the two possible tetragonal point groups (*P*4 and *P*422). While robust solutions were found in each round, the *P*4 run yielded a solution in space group *P*4_3_ with LLG = 117.2 and TFZ = 12.4, while the *P*422 run yielded a solution in space group *P*4_3_2_1_2 with LLG = 316.94 and TFZ = 21.0. The better of these solutions was consistent with the space group reported for every tetragonal lysozyme structure in the Protein Data Bank and was thus used in subsequent refinement.

For reasons of completeness, diffraction data to only 2.5 Å resolution were used for refinement, even though *I*/σ(*I*) remained high (>4) to 2.3 Å resolution and beyond. The structure was refined using *CNS* (Brunger, 2007 ▶) and *PHENIX* (Adams *et al.*, 2010 ▶), while manual rebuilding and adjustment were carried out in *Coot* (Emsley *et al.*, 2010 ▶). Despite the somewhat substantial X-ray dose received by each crystal at room temperature, no evidence of radiation damage could be seen in the MR-derived electron-density maps or the final refined structure. Final refinement statistics are shown in Table 1 ▶; although the *R*_work_ and *R*_free_ are somewhat higher than might be expected for HEWL at 2.5 Å resolution (27.2 and 30.7%, respectively), this modest inflation is likely to be owing to the challenges associated with reconstructing fully recorded reflections from still images and is expected to improve as the *PRIME* is developed further. All structural figures were produced in *PyMOL* (Schrödinger; http://www.schrodinger.com). The structure has been deposited in the Protein Data Bank (Berman *et al.*, 2000 ▶, 2003 ▶) as entry 4wmg.

## 3. Results   

### 3.1. A microfluidic trap array for harvesting protein microcrystals   

Although a number of microfluidic chips have been successfully designed to allow both the growth and analysis of protein crystals *in situ* (Gerdts *et al.*, 2008 ▶; Hansen *et al.*, 2006 ▶; Li *et al.*, 2006 ▶, 2010 ▶; Perry *et al.*, 2013 ▶; Sauter *et al.*, 2007 ▶; Soliman *et al.*, 2011 ▶; Zhu *et al.*, 2014 ▶; Heymann *et al.*, 2014 ▶), devices that collect and capture single crystals grown off-chip in a conventional format have not been described previously. By comparison, microfluidic devices have been successfully used to capture living cells from a solution and position them in regularly spaced traps (Chung *et al.*, 2011 ▶; Di Carlo *et al.*, 2006 ▶; Crane *et al.*, 2014 ▶; Tan & Takeuchi, 2007 ▶; Nilsson *et al.*, 2009 ▶; Skelley *et al.*, 2009 ▶). Cells trapped by such approaches are viable and capable of division and/or fusion (Chung *et al.*, 2011 ▶; Crane *et al.*, 2014 ▶; Di Carlo *et al.*, 2006 ▶; Skelley *et al.*, 2009 ▶), suggesting that a hydrodynamic trap array could be used to capture fragile macromolecular crystals and preserve their integrity for subsequent X-ray experiments.

**Figure 2:**
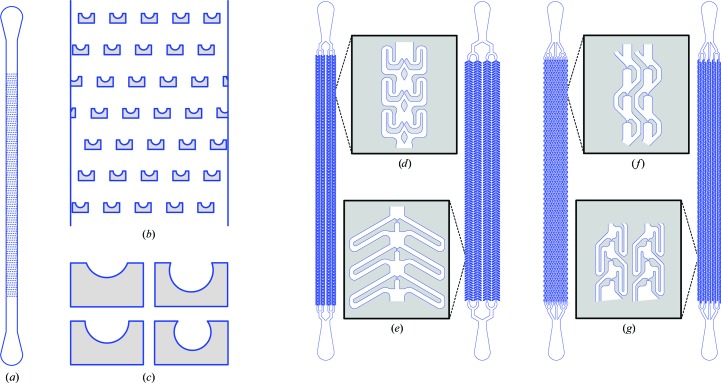
Trial designs of hydrodynamic trap arrays. (*a*, *b*, *c*) Prototype weir-type array design. (*a*) Overview of microfluidic device design. (*b*) Close-up of the post array inside a flow cell. (*c*) Comparison of a few of the different trap depths and geometries used for testing the weir-trap approach. (*d*, *e*, *f*, *g*) Prototype trap-and-bypass array device designs and close-ups of channel/trap arrays. (*d*, *e*) Example symmetric designs. (*f*, *g*) Example asymmetric designs. Asymmetric designs were found to capture crystals more reliably.

**Figure 3:**
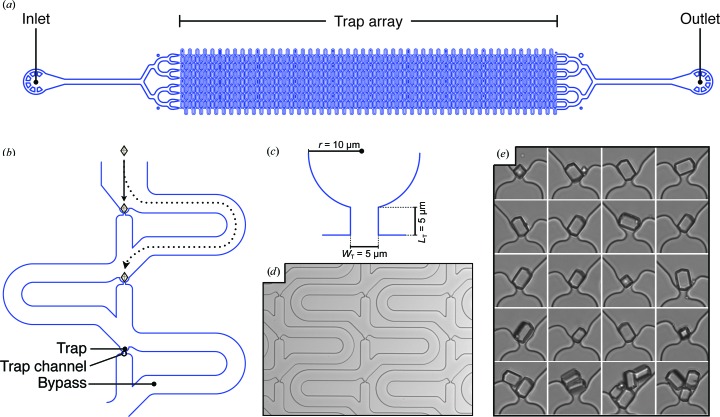
A microfluidic trap array for protein microcrystals. (*a*) Schematic representation of the crystal-capturing device design. (*b*) Close-up of the general scheme for trap-and-bypass hydrodynamic crystal capture. (*c*) Schematic of a single hydrodynamic trap [labeled in (*b*); *W*_T_ is the width of the trap channel and *L*_T_ is the length of the trap channel]. (*d*) Light micrograph of a representative section of a fabricated crystal-capture chip. (*e*) Light-micrograph series showing single and multiple HEWL microcrystals immobilized in hydrodynamic traps.

The simplest designs most amenable to adaptation for crystal trapping fall into two general categories: ‘weir’-type (Crane *et al.*, 2014 ▶; Di Carlo *et al.*, 2006 ▶; Skelley *et al.*, 2009 ▶) and trap-and-bypass (Chung *et al.*, 2011 ▶; Tan & Takeuchi, 2007 ▶). In a weir-type trap array, the flow cell is a wide channel with regularly spaced small posts that serve as traps (below page view in Figs. 2 ▶ *a*, 2 ▶ *b* and 2 ▶ *c*). The weir posts do not span the entire channel height, but leave a narrow gap under the post (Figs. 2 ▶ *a*, 2 ▶ *b* and 2 ▶ *c*) to increase the flow through the weir and to direct incoming particles into the trap. Trap-and-bypass schemes are constructed from a relatively narrow flow channel that is constricted at regular intervals and that contains a wider secondary bypass channel at each constriction point (Figs. 2 ▶ *d*, 2 ▶ *e*, 2 ▶ *f* and 2 ▶ *g*). During the operation of these devices, once a trap is obstructed by an incoming particle, subsequent particles are diverted into the bypass channel and directed towards the subsequent trap (Fig. 3 ▶ *b*).

We first tested a series of weir-type trap arrays, since they appeared to have the greater simplicity of design; the only variations between individual test designs were the size and shape of the trap cavity and the spacing between the traps (Fig. 2 ▶ *c*). During prototyping and testing, however, it became apparent that while simple to design, weir-type trap arrays were difficult to fabricate such that the gap under the posts was routinely large enough to allow the rapid flow of buffer solution yet sufficiently small to trap microcrystals (generally, crystals tended to flow through this gap and under the traps). Moreover, as has been noted for cell-trapping devices (Chung *et al.*, 2011 ▶; Skelley *et al.*, 2009 ▶), the weir-type arrays suffer from relatively low capture efficiency. This limitation can be improved to ∼70% by reducing the spacing between the traps to 20–50 µm (Skelley *et al.*, 2009 ▶), but the close spacing increases the risk of clogging. Reasoning that heat propagating from an XFEL pulse might affect such closely spaced traps, and thereby damage and waste valuable sample, we abandoned the weir-type arrays as a microcrystal-capture framework.

Trap-and-bypass arrays have been developed as a high-efficiency capture alternative to weir-type chips (Chung *et al.*, 2011 ▶). Starting from a single design that worked with spherical ∼15 µm sized microbeads and live human cells (Chung *et al.*, 2011 ▶), we sequentially modified the design by expanding the array to contain several hundred traps (instead of the original 64) and by eliminating a large compartment for cell growth (Figs. 2 ▶ *d*, 2 ▶ *e*, 2 ▶ *f* and 2 ▶ *g*). We also created and tested both symmetric (Figs. 2 ▶ *d* and 2 ▶ *e*) and asymmetric (Figs. 2 ▶ *f* and 2 ▶ *g*) variations of the array. Surprisingly, only asymmetric versions of the array were capable of trapping crystals. We speculate that this difference may arise from the path of the bypass channel, which in an asymmetric array directs a crystal that misses one trap immediately towards the next downstream trap (as opposed to a subsequent bypass channel, as occurs in the symmetric array).

After several rounds of testing and redesign to maximize crystal-capture efficiency and minimize clogging propensity (Figs. 3 ▶ *a* and 3 ▶ *b*), we arrived at a design in which a single inlet channel is divided into eight separate channels that each contain 100 traps (the channels then re-converge into a single outlet; Fig. 3 ▶ *a*). The trap channel was constructed to be sufficiently narrow to enable the capture of microcrystals, while the bypass channel is much wider, with smooth turns to prevent clogging (Fig. 3 ▶ *b*). Since the ratio of the volumetric flow through the trap and bypass channels directly influences the capture efficiency of each individual trap (Chung *et al.*, 2011 ▶), the trap and bypass channels were made as short and as long as reasonably attainable, respectively. Finally, we spaced the traps 150 × 300 µm apart so as to mitigate X-ray and thermal damage to adjacent crystals while maximizing the number of traps within the chip. The final chip design utilizes a semicircular trap 20 µm in diameter, with the trap channel constricting to 5 µm long and 5 µm wide (Fig. 3 ▶ *c*). The flow-channel height is set to 10 µm, providing a 1:2 aspect ratio to aid fabrication, while the bypass channels are 40 µm wide and ∼550 µm long. Because the bypass channels have been designed to interlock, 800 traps can be fitted into a 15 × 2.6 mm array. A light micrograph of a representative section of the fabricated chip is shown in Fig. 3 ▶(*d*).

### 3.2. Efficient capture of protein microcrystals in a hydrodynamic trap array   

We chose to use hen egg-white lysozyme (HEWL) crystals approximately 10–15 µm in the longest dimension for capture trials, as their location and orientation could be easily visualized by light microscopy. The bypass trap array proved very effective at efficiently capturing individual crystals from the microcrystalline slurry (Fig. 3 ▶ *e*). Visual observation of the loading process clearly reveals the capture mechanism in action as the traps fill in sequence from the inlet to outlet direction (Supplementary Movie S1). Pile-ups of multiple crystals occurred in some traps (Fig. 3 ▶ *e*, bottom row), presumably when the first trapped crystal left a portion of the trap channel unblocked, allowing other crystals to be directed into the same trap. On some occasions, typically when using crystals of >30 µm in length (which exceeded the design parameters of the chip), the high density of the incoming crystal stream led to clogging of the channels.

**Figure 4:**
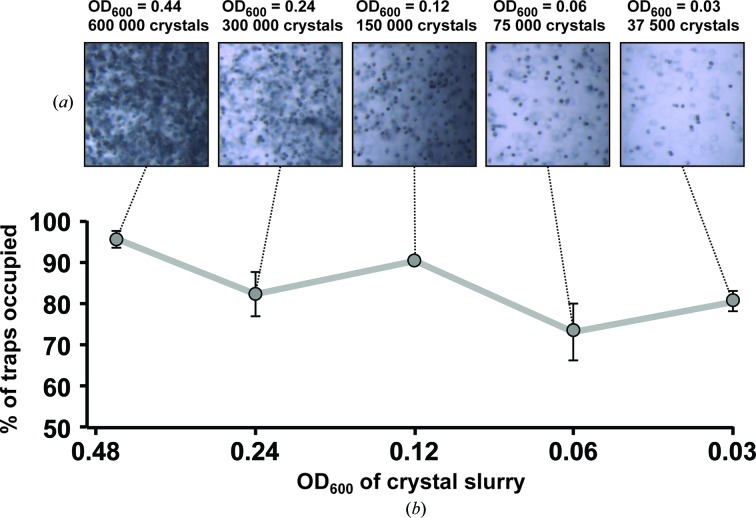
Crystal-capture efficiency in a hydrodynamic trap array. (*a*) A dilution series of microcrystalline HEWL slurry; the OD_600_ of the slurry and the estimated crystal count per load (5 µl) are indicated. (*b*) Percentage of traps filled in a single chip as a function of slurry concentration. Error bars represent the standard error calculated from a triplicate experiment.

To quantitate the actual capture efficiency of the trap array, we performed a series of loading experiments with microcrystalline lysozyme slurries of varying concentrations. We used measurements of the optical density at 600 nm (OD_600_) and combined them with visual inspection of the slurry so as to estimate the concentration of the microcrystalline slurry used for each experiment (Fig. 4 ▶ *a*). To determine the approximate number of crystals per loading experiment, we photographed a representative 1 µl drop of microcrystalline slurry, sectioned it into a grid and counted the number of crystals per grid square; multiplying this number by the number of squares in the grid yielded an approximate value of 120 000 HEWL microcrystals per microlitre of slurry in the most concentrated sample (OD_600_ = 0.44). From this starting point, we then applied serial twofold dilutions to generate a range of concentrations from OD_600_ = 0.44 to OD_600_ = 0.03 (Fig. 4 ▶ *a*).

Loading experiments with the dilution series showed robust capture efficiency even at the lowest assayed concentration, OD_600_ = 0.03 (Fig. 4 ▶ *b*). In virtually all cases more than 70% of the traps were occupied by one or more crystals, a value in excellent agreement with the theoretical capture efficiency (also 70%) calculated for the design (equations 1 and 2). Triplicate experiments revealed a modest variability in trapping efficiency, suggesting that operational details (for example, chip quality, handling, crystal transfer into tubing *etc.*) have an impact on the capture process. In our dilution-series loading experiment, 5 µl of slurry (corresponding to approximately 37 500–600 000 crystals, depending on the OD_600_) were injected into each chip. While this methodology ensured comparability of overall loading efficiency, in many cases a chip could be fully loaded with only 1–2 µl of slurry.

To gain a quantitative insight into the actual per-trap capturing efficiency of the chip, we also tested performance with an extremely dilute microcrystalline sample, such that the number of crystals injected into the chip would be much smaller than the total number of traps in the array (Table 2 ▶). This experiment was performed in order to ensure that empty traps would be available for each injected crystal in each of the eight channels and that every incoming crystal could be accounted for by visual inspection. The outcome of this study markedly exceeded the calculated per-trap capture efficiency: out of the 139 crystals injected, 120 were captured in 111 traps, representing an 86.3% efficiency in capturing individual crystals and an 79.9% efficiency in filling individual traps (Table 2 ▶; on a per-channel basis, capture efficiency varied from 75 to 100%). Interestingly, the inner channels received a greater flow of crystals than the outer channels, suggesting that larger sample volumes lead to slightly lower capture efficiencies owing to a greater number of crystals flowing through already populated inner channels. In this experiment, none of the injected crystals exited the chip; crystals that were not captured in traps were observed occupying the bypass channels.

### 3.3. X-ray diffraction of trapped microcrystals   

**Figure 5:**
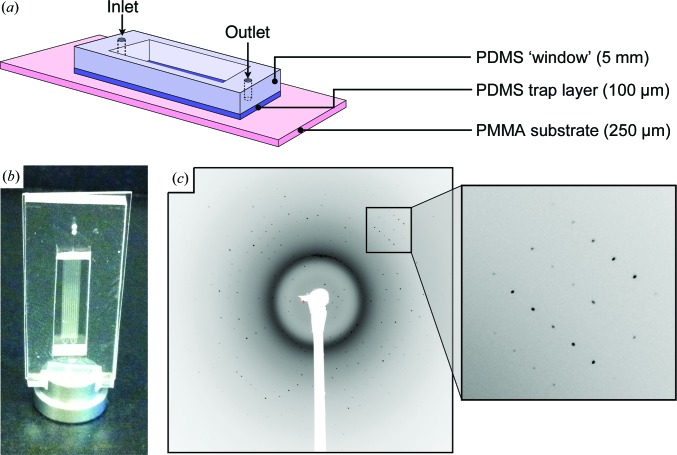
X-ray diffraction from and structural analysis of microcrystals captured in a hydrodynamic trap array. (*a*) Microfluidic crystal-trapping chip adapted for X-ray experiments. (*b*) Photograph of a complete chip coupled to a magnetic goniometer mount. (*c*) Representative diffraction image obtained from a trapped microcrystal using an XFEL pulse (inset: close-up of several reflections).

Having designed and implemented a robust microfluidic crystal-capture array, we next sought to evaluate the performance of the chips in X-ray diffraction experiments. To minimize both attenuation of the X-ray beam and the intrinsic scattering from PDMS (Dhouib *et al.*, 2009 ▶; Greaves & Manz, 2005 ▶; Guha *et al.*, 2012 ▶), we fabricated a hybrid chip with a 100 µm thick trap layer of PDMS and a 250 µm thick base of polymethyl methacrylate (PMMA; Fig. 5 ▶ *a*). PMMA was chosen both for its relatively high X-ray permeability and its lack of the scattering bands characteristic of PDMS and cyclic olefin co-polymer (COC; Dhouib *et al.*, 2009 ▶). A 2 mm thick PDMS window was placed around the trap array (Fig. 5 ▶ *a*) to stiffen the overall structure and to provide thickness for supporting the inlet and outlet tubing. Following fabrication, the thin trap-array chips were loaded with microcrystalline slurry at either a synchrotron or an XFEL beamline, attached to a standard magnetic base (Fig. 5 ▶ *b*) and mounted at room temperature for data collection.

**Figure 6:**
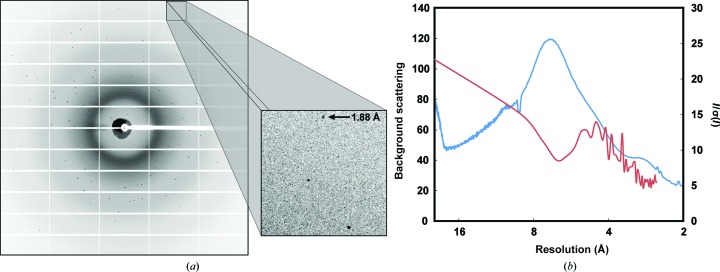
Microfocus diffraction and PDMS scattering. (*a*) Representative diffraction image obtained from a single trapped microcrystal using a 5 s exposure to synchrotron radiation and a 0.02° oscillation (inset: close-up of reflections with a high-resolution, 1.88 Å, reflection indicated). (*b*) Overlay of background scattering (blue) and *I*/σ(*I*) (red) *versus* resolution. The peak of the PDMS scattering ring is evident at 7.5 Å.

Hen egg-white lysozyme microcrystals ∼15 µm in size that were captured in the trap-array chip diffracted readily when exposed to X-ray beams from both (microfocus) synchrotron and XFEL light sources (Figs. 5 ▶ *c* and 6 ▶ *a*). While diffraction quality varied from crystal to crystal, strong reflections extending beyond 1.8 Å resolution could be observed in experiments conducted at either light source (Figs. 5 ▶ *c* and 6 ▶ *a*). Resolution to this extent is in line with XFEL diffraction data collected previously from native (unfrozen) HEWL microcrystals (∼5 µm) *in vacuo* and at room temperature (Boutet *et al.*, 2012 ▶). When indexed, both XFEL- and synchrotron-derived images yielded crystal parameters characteristic of the tetragonal HEWL crystal morphology, belonging to space group *P*4_3_2_1_2 and with unit-cell parameters *a* = *b* = 79.3, *c* = 38.2 Å, α = β = γ = 90° (Table 1 ▶). As anticipated from prior work using PDMS (Dhouib *et al.*, 2009 ▶; Hansen *et al.*, 2002 ▶), a characteristic scattering ring from this material at 7.5 Å was also observed in all images (Fig. 6 ▶ *b*).

### 3.4. Structure of hen egg-white lysozyme determined from single shots of trapped microcrystals   

Insufficient XFEL beamtime was available to collect a complete data set from HEWL crystals trapped in microfluidic chips. However, test images (collected using a 3 µm beam size at the XPP endstation of the LCLS) revealed that the chip-trapped HEWL microcrystals diffracted to a comparable resolution as seen in our microfocus studies (Fig. 5 ▶ *c*). Thus, to determine whether the crystal-capture chips could permit the collection of diffraction data suitable for structure determination, we collected a series of images on a microfocus synchrotron beamline (SSRL beamline 12-2). To simulate an XFEL-like experiment, we collected a single image per trapped crystal. Each crystal was selected by visual inspection of the chip and targeted manually. Since the trapped lysozyme crystals appeared to preferentially adopt a somewhat narrow range of orientations (Fig. 3 ▶ *e*), we randomly tilted the chip by angles ranging from 0 to 30° for different shots to increase positional diversity. Only one diffraction image was collected per targeted crystal. 265 diffraction images were collected in total, of which 232 could be integrated. The estimated resolution varied greatly from image to image, indicating a high variability of crystal quality in this batch of microcrystalline material (Supplementary Fig. S1 and Supplementary Table S1).

The combination of the trapped crystals and tilting of the chip served to sufficiently randomize the crystal orientation to allow the collection of a reasonably complete data set with relatively few exposures (265 diffraction images) on a microfocus synchrotron beamline (SSRL beamline 12-2; Fig. 6 ▶ *a*). The smallest available oscillation of 0.02° on the goniometer was used to approximate a zero-oscillation (‘still’) image typically obtained from a single pulse of an XFEL beam. Images were integrated with the *cctbx.xfel* suite of software (Hattne *et al.*, 2014 ▶) and then merged and post-refined with *PRIME* (Uervirojnangkoorn *et al.*, 2015 ▶) to generate a data set of sufficient quality for structure determination (Table 1 ▶). We estimate that data sets could similarly be collected from lower-symmetry crystal forms using 1–2 trap-array devices, particularly if data collection were carried out in conjunction with tilted exposures.

**Figure 7:**
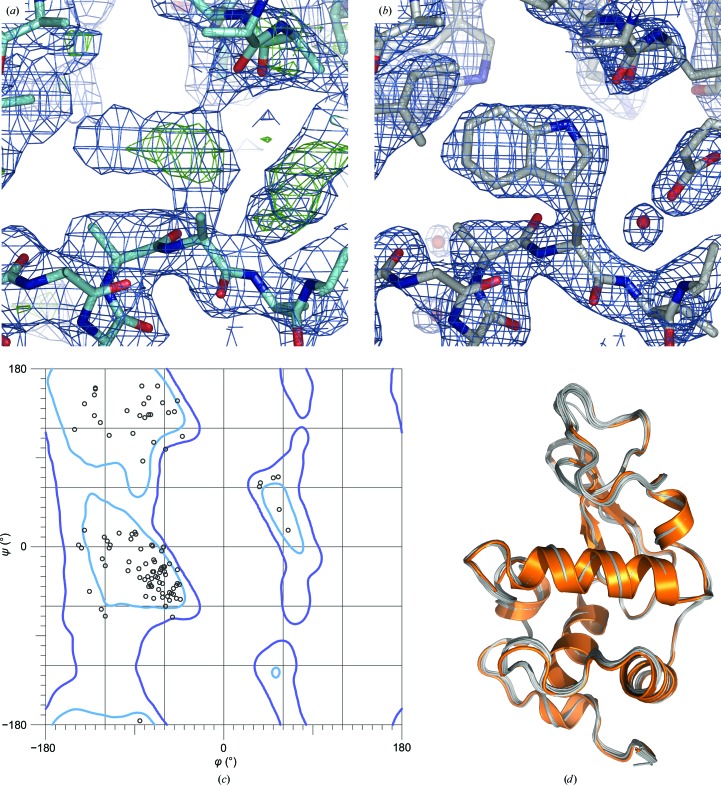
Solution and refinement of a tetragonal HEWL structure. (*a*) Electron-density maps (2*F*_o_ − *F*_c_ at 1.0σ, blue mesh; *F*_o_ − *F*_c_ at 2.5σ, green mesh) obtained from molecular replacement using a polyalanine model of tetragonal HEWL. (*b*) Final refined 2*F*_o_ − *F*_c_ map (1.5σ, blue mesh) of tetragonal HEWL at 2.5 Å resolution. (*c*) Ramachandran diagram of all amino-acid residues in the asymmetric unit. (*d*) Superposition of the tetragonal HEWL structure determined from in-chip diffraction data (orange) *versus * 15 tetragonal HEWL structures randomly drawn from the Protein Data Bank (grey).

Following diffraction data indexing, integration and post-refinement, phases for the HEWL structure were readily obtained by molecular replacement (MR). We used a search model generated from a structure of HEWL solved using data collected at room temperature (Pinker *et al.*, 2013 ▶) from which all water molecules, ligands and side chains had been removed. From this model, molecular replacement with *Phaser* (McCoy, 2007 ▶) generated a robust single solution with a log-likelihood gain of 310.7 and a *Z*-score of 20.4. Importantly, positive difference density was readily apparent for the side chains and disulfide linkages omitted from the model (Fig. 7 ▶ *a*). After six rounds of refinement and manual rebuilding, *R*_work_ and *R*_free_ converged to 27.2 and 30.7%, respectively, over the resolution range 23–2.5 Å, with good geometry and well resolved electron density (Figs. 7 ▶ *b* and 7 ▶ *c*); as per the validation tools implemented in *PHENIX* (Urzhumtseva *et al.*, 2009 ▶), these and all other resultant metrics were within standard ranges for this resolution. Overall, the structure very closely matches other HEWL structures obtained at room temperature (Fig. 7 ▶ *d*).

## 4. Discussion   

Although X-ray crystallography is a powerful method for determining three-dimensional structures of biological macromolecules at high resolution, it is inherently limited by a need for crystals of an intended target. It is not uncommon for crystal screening efforts to produce only (sub-)micrometre crystals, which in many instances can be refractory to optimization and/or harvesting. Even if larger crystals become available, methods of preserving the crystals for later data collection by flash-cooling are often laborious and may increase the crystal mosaicity or damage the crystal. To circumvent these issues and to efficiently deliver a set of pre-grown microcrystals to the X-ray beam of synchrotron or XFEL light source, we developed a microfluidic crystal-trapping chip. Using this device, we were able to efficiently capture microcrystals injected from a crystal slurry into a hydrodynamic trap array, collect X-ray diffraction images from the crystals using synchrotron and XFEL sources, and determine the structure of hen egg-white lysozyme from a set of serially collected pseudo-still images obtained at a synchrotron source.

The use of microfluidic chips in serial crystallography has been made possible by recent advances in data-processing software (Uervirojnangkoorn *et al.*, 2015 ▶). The program *PRIME* scales, merges and iteratively post-refines the integrated diffraction images, greatly improving the quality of the merged diffraction data set. This iterative post-refinement approach reduces limitations owing to the serial crystallography data-collection strategy, whereby only one still image is collected from each crystal. *PRIME* iteratively adjusts for the diffraction-quality difference owing to crystal size variation, refines crystal orientation parameters, refines unit-cell parameters and rejects outlier images and reflections. As a result, a complete data set can be assembled from many fewer diffraction images compared with the Monte Carlo summation used in the first pioneering XFEL crystallography studies (Kirian *et al.*, 2010 ▶; Chapman *et al.*, 2011 ▶). This approach in turn ensures that a complete data set may be obtainable from crystals captured in a single 800-trap microfluidic chip.

At present, a wide variety of microfluidic systems have been developed for the purposes of X-ray data collection from crystals grown *in situ* (Sauter *et al.*, 2007 ▶). These include droplet-based nanobatch devices (Gerdts *et al.*, 2008 ▶; Li *et al.*, 2006 ▶), including a recent design that allows the growth of a single crystal per droplet (Heymann *et al.*, 2014 ▶), free-interface diffusion (FID) chips (Hansen *et al.*, 2002 ▶, 2006 ▶; Perry *et al.*, 2013 ▶), FID/microbatch hybrids (Li *et al.*, 2010 ▶) and microfluidic adaptations of classic microbatch or vapor-diffusion plates (Soliman *et al.*, 2011 ▶; Zhu *et al.*, 2014 ▶). Many of these devices have been constructed to permit data collection from the device itself, eliminating the need for potentially disruptive crystal manipulation and enabling the collection of diffraction data at room temperature. However, none of these devices allow the analysis of samples grown by conventional, off-chip vapor-diffusion or batch methods, and few are capable of efficiently immobilizing microcrystals in fixed, addressable positions that can be automatically targeted.

The crystal-trapping chips described here represent an effective and efficient alternative means of delivering microcrystalline samples derived from any source or crystallization format to an XFEL or microfocus source. Notably, the trap-array chips consume a very low amount of crystal slurry (∼5 µl) and can be readily mounted on any standard gonio­meter. A second powerful attribute of the chips is that they immobilize crystals in fixed positions along a regular array, allowing automated targeting to provide a near-100% chance of hitting each crystal with the X-ray beam. Finally, the crystals are held in microchannels filled with the crystallization buffer, allowing diffraction data to be collected at room temperature. This latter feature, which circumvents the need for complex and potentially damaging *in vacuo* and cryo techniques, enables researchers to take full advantage of the ‘diffraction-before-destruction’ capability of XFELs while potentially identifying structural substates that can disappear upon flash-cooling (Fraser *et al.*, 2009 ▶, 2011 ▶).

As is typical for soft polymer microfluidic devices in general, the crystal-trapping chips described here are inexpensive and easy to fabricate. Another attractive aspect of using microfluidic capturing devices is the versatility of the format. The channel and trap dimensions can be easily altered to accommodate crystals of a variety of sizes. Furthermore, the relatively simple, modular design allows the harvesting trap array to be paired with a wide variety of micro-crystallization approaches, including setups used to generate microcrystals of small molecules. The chips also could be further outfitted with microfluidic valves capable of sequestering individual crystals in soaking chambers, for example as a means to carry out ligand or heavy-metal soaks and screening *in situ*. Finally, the scalability of the trap array allows the chips to be made larger or smaller to better accommodate the setup constraints of individual beamlines.

A drawback endemic to using PDMS-based microfluidic devices for diffraction studies is their X-ray absorption and scattering properties (Guha *et al.*, 2012 ▶; Greaves & Manz, 2005 ▶; Dhouib *et al.*, 2009 ▶). Despite employing a relatively thin (90 µm) PDMS layer for the X-ray-exposed portion of the trap array, we still observed strong scattering at 7.5 Å (Figs. 5 ▶ *c* and 6 ▶), a characteristic of diffraction obtained using PDMS-based devices (Guha *et al.*, 2012 ▶; Hansen *et al.*, 2006 ▶); accordingly, there is a decrease in diffraction data quality [*I*/σ(*I*)] that correlates with the PDMS-related scattering (Fig. 6 ▶ *b*). Thus, while very useful for fast prototyping, PDMS may not be the ideal material for routine X-ray diffraction studies. As a workaround, the trapping devices described here could be constructed from more X-ray-permeable materials such as PMMA, cyclic olefin co-polymer (COC) or silicon nitride (Dhouib *et al.*, 2009 ▶; Guha *et al.*, 2012 ▶; Hunter *et al.*, 2014 ▶). The relative simplicity of the trap array (single flow layer, no valves) should enable the transition from PDMS to other materials or microfabrication techniques (*e.g.* hot embossing or wet etching) with modest effort. Development in several of these directions is currently under way.

## Supplementary Material

PDB reference: hen egg-white lysozyme, 4wmg

Supplementary Movie S1. Hydrodynamic capture of lysozyme microcrystals in a microfluidic trap array.. DOI: 10.1107/S1399004715002308/wa5087sup1.mov

Supplementary Figure 1 and Table 1, showing variation in diffraction resolution on an image-by-image basis.. DOI: 10.1107/S1399004715002308/wa5087sup2.pdf

## Figures and Tables

**Table 1:** Data-collection and refinement statistics Values in parentheses are for the highest resolution shell.

Data-collection and merging statistics	
Wavelength ()	0.98
Resolution range ()	21.982.50 (2.542.50)
Space group	*P*4_3_2_1_2
Unit-cell parameters (, )	*a* = *b* = 79.30, *c* = 38.12, = = = 90.0
Total reflections	22965
Unique reflections	4520 (227)
No. of observations	5.1 (1.90)
Completeness (%)	83.0 (67.8)
Mean *I*/(*I*)	9.0 (6.15)
Wilson *B* factor (^2^)	32.77
CC_1/2_ (%)	88.1 (16.51)
Structure-refinement statistics	
*R*_work_	0.272
*R*_free_	0.307
R.m.s.d., bonds ()	0.004
R.m.s.d., angles ()	0.85
Ramachandran favored (%)	86.0
Ramachandran allowed (%)	14.0
Ramachandran outliers (%)	0

**Table 2:** Low-concentration crystal-loading experiment

Channel	Injected	Traps filled	Crystals trapped	Traps filled (%)	Crystals trapped (%)
1	2	0	0	0.0	0.0
2	10	7	8	70.0	80.0
3	18	17	18	94.4	100.0
4	42	30	35	71.4	83.3
5	45	37	38	82.2	84.4
6	18	17	18	94.4	100.0
7	4	3	3	75.0	75.0
8	0	0	0	0.0	0.0
Total	139	111	120	79.9	86.3
