# Supplementary material for: Capture and X-ray diffraction studies of protein microcrystals in a microfluidic trap array
Source: Acta Crystallogr D Biol Crystallogr. 2015 Mar 27;71(Pt 4):928–40. doi: 10.1107/S1399004715002308 (PMC4388268; doi:10.1107/S1399004715002308)
Supplement: Supplementary file 2 [file d-71-00928-sup2.pdf]

BIOLOGICAL CRYSTALLOGRAPHY

Volume 71 (2015)

Supporting information for article:

Capture and X-ray diffraction studies of protein microcrystals in a microfluidic trap array

Artem Y. Lyubimov, Thomas D. Murray, Antoine Koehl, Ismail Emre Araci, Monarin Uervirojnangkoorn, Oliver B. Zeldin, Aina E. Cohen, S. Michael Soltis, Elizabeth L. Baxter, Aaron S. Brewster, Nicholas K. Sauter, Axel T. Brunger and James M. Berger

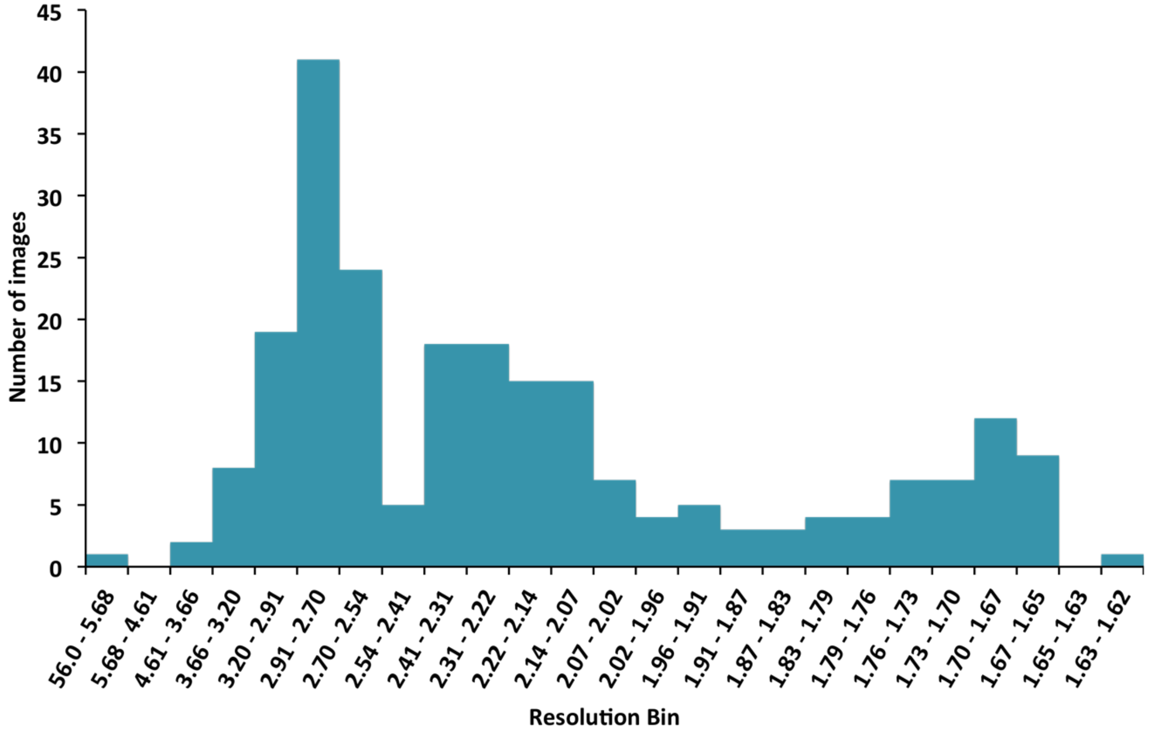

Supplemental Figure 1. Histogram of the limiting resolution of individual diffraction images using 232 successfully integrated diffraction images collected from in-chip HEWL microcrystals. After scaling, merging and post-refinement, the overall resolution limit was truncated to 2.5 Å, which approximately corresponds to the mean of the resolution limits obtained from the individual diffraction images.

Supplemental Table 1. Space group, unit cell and limiting resolution of individual integrated diffraction images collected from in-chip HEWL microcrystals.

Image #	Space group	a	b	c	α	β	γ	Resolution
**1**	P 4	78.71	78.71	38.69	90	90	90	38.69 - 2.74
**2**	P 4	79.78	79.78	38.02	90	90	90	15.76 - 2.06
**3**	P 4	79.96	79.96	38.05	90	90	90	17.88 - 2.79
**4**	P 4	79.67	79.67	38.02	90	90	90	56.33 - 2.56
**5**	P 4	79.77	79.77	38.01	90	90	90	38.01 - 2.81
**6**	P 4	80.37	80.37	38.03	90	90	90	11.58 - 4.21
**7**	P 4	79.95	79.95	38.04	90	90	90	56.53 - 2.22
**8**	P 4	80.00	80.00	38.06	90	90	90	56.57 - 2.74
**9**	P 4	80.18	80.18	37.98	90	90	90	31.55 - 2.93
**10**	P 4	80.43	80.43	38.04	90	90	90	38.04 - 3.29
**11**	P 4	80.19	80.19	37.85	90	90	90	22.24 - 2.75
**12**	P 4	80.04	80.04	37.95	90	90	90	37.95 - 2.33
**13**	P 4	79.99	79.99	37.96	90	90	90	56.56 - 2.27
**14**	P 4	80.09	80.09	38.00	90	90	90	28.32 - 3.61

Image #	Space group	a	b	c	α	β	γ	Resolution
**15**	P 4	80.00	80.00	37.99	90	90	90	37.99 - 2.82
**16**	P 4	80.06	80.06	38.06	90	90	90	56.61 - 2.21
**17**	P 4	79.90	79.90	38.00	90	90	90	56.50 - 2.88
**18**	P 4	80.75	80.75	38.15	90	90	90	21.99 - 2.97
**19**	P 4	80.32	80.32	37.93	90	90	90	37.93 - 2.76
**20**	P 4	80.07	80.07	37.91	90	90	90	12.48 - 4.26
**21**	P 4	80.21	80.21	37.92	90	90	90	10.27 - 2.88
**22**	P 4	79.27	79.27	38.12	90	90	90	38.12 - 1.73
**23**	P 4	79.41	79.41	38.09	90	90	90	15.57 - 1.80
**24**	P 4	79.56	79.56	38.12	90	90	90	38.12 - 2.06
**25**	P 4	79.74	79.74	38.04	90	90	90	79.74 - 2.28
**26**	P 4	79.71	79.71	38.08	90	90	90	56.36 - 2.26
**27**	P 4	79.73	79.73	38.06	90	90	90	56.38 - 2.66
**28**	P 4	79.78	79.78	38.04	90	90	90	38.04 - 2.26
**29**	P 4	79.92	79.92	38.05	90	90	90	31.56 - 2.68
**30**	P 4	79.91	79.91	38.08	90	90	90	56.51 - 2.61
**31**	P 4	79.21	79.21	38.16	90	90	90	38.16 - 1.66
**32**	P 4	80.55	80.55	37.91	90	90	90	12.66 - 2.94
**33**	P 4	79.88	79.88	38.03	90	90	90	38.03 - 3.62
**34**	P 4	79.96	79.96	38.02	90	90	90	11.34 - 3.25
**35**	P 4	79.91	79.91	38.03	90	90	90	56.51 - 2.86
**36**	P 4	80.15	80.15	37.96	90	90	90	56.67 - 3.35
**37**	P 4	79.89	79.89	37.95	90	90	90	56.49 - 2.67
**38**	P 4	79.98	79.98	37.96	90	90	90	37.96 - 3.02
**39**	P 4	79.66	79.66	38.00	90	90	90	18.78 - 2.76
**40**	P 4	79.40	79.40	38.00	90	90	90	56.15 - 2.63
**41**	P 4	80.18	80.18	37.90	90	90	90	37.90 - 3.34
**42**	P 4	80.03	80.03	37.90	90	90	90	10.32 - 2.41
**43**	P 4	79.85	79.85	37.97	90	90	90	56.46 - 2.87
**44**	P 4	80.13	80.13	36.86	90	90	90	25.34 - 2.94
**45**	P 4	79.92	79.92	37.94	90	90	90	31.50 - 2.98
**46**	P 4	79.92	79.92	38.08	90	90	90	56.51 - 2.88
**47**	P 4	80.21	80.21	38.04	90	90	90	22.74 - 2.54
**48**	P 4	80.13	80.13	37.99	90	90	90	16.03 - 3.60
**49**	P 4	79.65	79.65	38.11	90	90	90	18.05 - 2.68
**50**	P 4	80.40	80.40	37.95	90	90	90	18.98 - 1.86
**51**	P 4	79.77	79.77	38.25	90	90	90	56.41 - 2.98
**52**	P 4	79.94	79.94	38.03	90	90	90	17.88 - 2.71
**53**	P 4	79.89	79.89	37.96	90	90	90	12.22 - 3.25
**54**	P 4	79.68	79.68	38.04	90	90	90	38.04 - 1.79
**55**	P 4	79.49	79.49	38.11	90	90	90	38.11 - 2.29

Image #	Space group	a	b	c	α	β	γ	Resolution		
**56**	P 4	79.91	79.91	38.09	90	90	90	22.16	-	2.72
**57**	P 4	79.52	79.52	38.19	90	90	90	56.23	-	2.37
**58**	P 4	79.63	79.63	38.06	90	90	90	38.06	-	2.40
**59**	P 4	79.77	79.77	38.07	90	90	90	31.55	-	1.76
**60**	P 4	79.83	79.83	38.05	90	90	90	18.03	-	2.66
**61**	P 4	79.96	79.96	38.04	90	90	90	56.54	-	2.58
**62**	P 4	80.24	80.24	38.09	90	90	90	19.22	-	2.89
**63**	P 4	79.11	79.11	38.12	90	90	90	55.94	-	1.66
**64**	P 4	79.05	79.05	38.09	90	90	90	16.74	-	2.34
**65**	P 4	79.06	79.06	38.19	90	90	90	25.94	-	1.72
**66**	P 4	79.32	79.32	38.11	90	90	90	79.32	-	2.05
**67**	P 4	79.27	79.27	38.19	90	90	90	31.56	-	2.38
**68**	P 4	78.79	78.79	38.42	90	90	90	19.00	-	2.16
**69**	P 4	78.83	78.83	38.55	90	90	90	31.71	-	2.76
**70**	P 4	78.97	78.97	38.14	90	90	90	34.34	-	2.05
**71**	P 4	79.09	79.09	38.13	90	90	90	27.45	-	2.88
**72**	P 4	78.76	78.76	38.18	90	90	90	14.63	-	2.38
**73**	P 4	79.18	79.18	38.09	90	90	90	55.99	-	1.69
**74**	P 4	79.33	79.33	38.15	90	90	90	31.54	-	1.89
**75**	P 4	79.23	79.23	38.07	90	90	90	22.56	-	1.73
**76**	P 4	79.30	79.30	38.12	90	90	90	16.79	-	1.76
**77**	P 4	79.25	79.25	38.17	90	90	90	56.04	-	1.94
**78**	P 4	79.32	79.32	38.17	90	90	90	34.40	-	2.12
**79**	P 4	79.40	79.40	38.16	90	90	90	19.26	-	2.72
**80**	P 4	78.92	78.92	38.29	90	90	90	55.80	-	2.85
**81**	P 4	79.08	79.08	38.15	90	90	90	18.54	-	1.65
**82**	P 4	79.25	79.25	38.18	90	90	90	16.07	-	1.68
**83**	P 4	79.38	79.38	38.14	90	90	90	16.80	-	1.66
**84**	P 4	79.29	79.29	38.13	90	90	90	20.95	-	1.69
**85**	P 4	79.10	79.10	38.25	90	90	90	19.03	-	2.29
**86**	P 4	79.37	79.37	38.12	90	90	90	22.01	-	1.73
**87**	P 4	80.05	80.05	38.03	90	90	90	56.60	-	2.60
**88**	P 4	79.60	79.60	38.10	90	90	90	56.29	-	2.27
**89**	P 4	79.06	79.06	38.09	90	90	90	55.91	-	2.17
**90**	P 4	79.17	79.17	38.07	90	90	90	31.48	-	1.66
**91**	P 4	78.71	78.71	38.24	90	90	90	17.60	-	2.59
**92**	P 4	79.37	79.37	38.20	90	90	90	31.58	-	2.06
**93**	P 4	78.64	78.64	38.66	90	90	90	38.66	-	2.31
**94**	P 4	79.72	79.72	38.15	90	90	90	56.37	-	2.98
**95**	P 4	79.78	79.78	38.01	90	90	90	56.41	-	2.78
**96**	P 4	79.41	79.41	38.13	90	90	90	38.13	-	1.78

Image #	Space group	a	b	c	α	β	γ	Resolution		
**97**	P 4	79.22	79.22	38.28	90	90	90	31.60	-	1.80
**98**	P 4	78.96	78.96	38.10	90	90	90	20.89	-	2.31
**99**	P 4	79.16	79.16	38.18	90	90	90	55.97	-	1.68
**100**	P 4	79.51	79.51	38.12	90	90	90	34.37	-	2.34
**101**	P 4	78.71	78.71	38.83	90	90	90	11.13	-	3.06
**102**	P 4	79.67	79.67	38.01	90	90	90	27.50	-	2.27
**103**	P 4	79.23	79.23	38.15	90	90	90	10.75	-	1.93
**104**	P 4	79.26	79.26	38.19	90	90	90	22.59	-	2.01
**105**	P 4	79.33	79.33	38.10	90	90	90	56.10	-	2.19
**106**	P 4	78.78	78.78	38.38	90	90	90	55.71	-	2.61
**107**	P 4	79.34	79.34	38.17	90	90	90	12.99	-	2.07
**108**	P 4	79.30	79.30	38.08	90	90	90	19.04	-	2.08
**109**	P 4	79.27	79.27	38.12	90	90	90	34.36	-	2.10
**110**	P 4	79.38	79.38	38.17	90	90	90	26.00	-	1.68
**111**	P 4	80.20	80.20	38.20	90	90	90	38.20	-	5.68
**112**	P 4	79.35	79.35	38.22	90	90	90	15.20	-	1.71
**113**	P 4	79.33	79.33	38.11	90	90	90	79.33	-	1.74
**114**	P 4	79.37	79.37	38.14	90	90	90	38.14	-	1.92
**115**	P 4	79.64	79.64	38.40	90	90	90	34.59	-	2.20
**116**	P 4	79.11	79.11	38.16	90	90	90	55.94	-	1.69
**117**	P 4	79.90	79.90	38.06	90	90	90	38.06	-	2.13
**118**	P 4	79.58	79.58	38.07	90	90	90	38.07	-	2.64
**119**	P 4	79.31	79.31	38.12	90	90	90	19.05	-	1.65
**120**	P 4	79.42	79.42	38.12	90	90	90	31.54	-	2.66
**121**	P 4	79.31	79.31	38.11	90	90	90	56.08	-	1.96
**122**	P 4	79.11	79.11	38.17	90	90	90	38.17	-	1.87
**123**	P 4	78.94	78.94	38.27	90	90	90	55.82	-	1.68
**124**	P 4	78.98	78.98	38.19	90	90	90	55.85	-	2.18
**125**	P 4	79.44	79.44	38.10	90	90	90	15.89	-	1.71
**126**	P 4	79.01	79.01	38.26	90	90	90	38.26	-	2.10
**127**	P 4	78.72	78.72	38.44	90	90	90	55.66	-	1.70
**128**	P 4	78.55	78.55	38.41	90	90	90	38.41	-	2.15
**129**	P 4	78.75	78.75	38.61	90	90	90	38.61	-	2.20
**130**	P 4	78.54	78.54	38.38	90	90	90	55.53	-	2.11
**131**	P 4	79.34	79.34	38.11	90	90	90	38.11	-	1.69
**132**	P 4	79.53	79.53	38.11	90	90	90	34.37	-	1.91
**133**	P 4	79.30	79.30	38.09	90	90	90	79.30	-	2.62
**134**	P 4	79.11	79.11	38.18	90	90	90	38.18	-	1.70
**135**	P 4	79.35	79.35	38.07	90	90	90	38.07	-	2.20
**136**	P 4	78.93	78.93	38.46	90	90	90	55.81	-	2.85
**137**	P 4	79.20	79.20	38.11	90	90	90	38.11	-	2.83

Image #	Space group	a	b	c	α	β	γ	Resolution
**138**	P 4	79.67	79.67	38.04	90	90	90	38.04 - 2.88
**139**	P 4	79.99	79.99	38.14	90	90	90	79.99 - 2.77
**140**	P 4	79.50	79.50	38.09	90	90	90	79.50 - 1.74
**141**	P 4	79.68	79.68	38.09	90	90	90	13.57 - 2.79
**142**	P 4	79.76	79.76	38.16	90	90	90	56.40 - 2.28
**143**	P 4	79.31	79.31	38.22	90	90	90	56.08 - 2.39
**144**	P 4	79.93	79.93	38.11	90	90	90	15.80 - 2.71
**145**	P 4	79.56	79.56	38.43	90	90	90	22.07 - 2.92
**146**	P 4	79.54	79.54	38.32	90	90	90	56.24 - 3.12
**147**	P 4	79.88	79.88	38.17	90	90	90	56.48 - 2.30
**148**	P 4	79.71	79.71	38.14	90	90	90	56.37 - 2.78
**149**	P 4	79.10	79.10	38.11	90	90	90	38.11 - 2.49
**150**	P 4	80.10	80.10	38.05	90	90	90	38.05 - 2.69
**151**	P 4	80.14	80.14	38.06	90	90	90	56.67 - 2.83
**152**	P 4	80.23	80.23	38.05	90	90	90	22.74 - 2.80
**153**	P 4	78.95	78.95	38.34	90	90	90	16.74 - 2.13
**154**	P 4	78.65	78.65	38.46	90	90	90	13.35 - 1.65
**155**	P 4	78.89	78.89	38.25	90	90	90	55.78 - 1.95
**156**	P 4	78.95	78.95	38.36	90	90	90	35.31 - 2.76
**157**	P 4	78.74	78.74	38.46	90	90	90	38.46 - 1.68
**158**	P 4	78.71	78.71	38.33	90	90	90	55.66 - 2.02
**159**	P 4	79.40	79.40	38.41	90	90	90	56.14 - 2.79
**160**	P 4	79.09	79.09	38.53	90	90	90	38.53 - 2.68
**161**	P 4	79.73	79.73	38.11	90	90	90	19.13 - 2.53
**162**	P 4	78.85	78.85	38.28	90	90	90	18.60 - 2.00
**163**	P 4	79.20	79.20	38.08	90	90	90	31.49 - 1.62
**164**	P 4	78.97	78.97	38.45	90	90	90	55.84 - 3.04
**165**	P 4	79.12	79.12	38.11	90	90	90	55.94 - 2.10
**166**	P 4	79.23	79.23	38.19	90	90	90	22.59 - 2.79
**167**	P 4	79.57	79.57	38.14	90	90	90	22.64 - 2.14
**168**	P 4	79.16	79.16	38.29	90	90	90	39.58 - 2.28
**169**	P 4	79.24	79.24	39.04	90	90	90	39.04 - 1.66
**170**	P 4	79.30	79.30	38.28	90	90	90	56.08 - 2.24
**171**	P 4	79.71	79.71	38.21	90	90	90	18.79 - 2.67
**172**	P 4	79.36	79.36	38.34	90	90	90	31.66 - 3.11
**173**	P 4	79.71	79.71	38.09	90	90	90	15.94 - 2.12
**174**	P 4	80.02	80.02	38.06	90	90	90	56.59 - 2.93
**175**	P 4	79.71	79.71	38.01	90	90	90	34.31 - 2.81
**176**	P 4	80.14	80.14	38.19	90	90	90	34.48 - 3.19
**177**	P 4	79.85	79.85	38.13	90	90	90	79.85 - 2.63
**178**	P 4	79.99	79.99	38.01	90	90	90	38.01 - 2.21

Image #	Space group	a	b	c	α	β	γ	Resolution		
**179**	P 4	79.91	79.91	37.96	90	90	90	37.96	-	2.66
**180**	P 4	78.97	78.97	38.29	90	90	90	38.29	-	2.91
**181**	P 4	78.86	78.86	38.31	90	90	90	38.31	-	2.50
**182**	P 4	79.00	79.00	38.31	90	90	90	55.86	-	2.69
**183**	P 4	78.71	78.71	38.51	90	90	90	22.56	-	2.27
**184**	P 4	79.22	79.22	38.32	90	90	90	38.32	-	2.70
**185**	P 4	78.06	78.06	38.81	90	90	90	38.81	-	1.70
**186**	P 4	78.33	78.33	38.87	90	90	90	15.29	-	3.17
**187**	P 4	79.06	79.06	38.31	90	90	90	38.31	-	1.87
**188**	P 4	78.51	78.51	38.77	90	90	90	55.51	-	1.84
**189**	P 4	78.49	78.49	38.72	90	90	90	34.73	-	2.03
**190**	P 4	78.46	78.46	38.59	90	90	90	13.05	-	1.75
**191**	P 4	79.07	79.07	38.52	90	90	90	55.91	-	2.86
**192**	P 4	79.16	79.16	38.29	90	90	90	55.98	-	2.38
**193**	P 4	78.52	78.52	38.70	90	90	90	27.56	-	2.12
**194**	P 4	79.71	79.71	38.22	90	90	90	56.36	-	2.94
**195**	P 4	78.58	78.58	38.47	90	90	90	27.49	-	3.14
**196**	P 4	78.59	78.59	38.39	90	90	90	25.92	-	2.70
**197**	P 4	79.93	79.93	38.23	90	90	90	38.23	-	2.70
**198**	P 4	78.48	78.48	38.48	90	90	90	18.18	-	1.65
**199**	P 4	78.76	78.76	38.46	90	90	90	20.91	-	2.28
**200**	P 4	79.58	79.58	38.22	90	90	90	56.27	-	2.84
**201**	P 4	78.85	78.85	38.33	90	90	90	16.84	-	2.24
**202**	P 4	78.55	78.55	38.58	90	90	90	38.58	-	2.34
**203**	P 4	78.66	78.66	38.43	90	90	90	38.43	-	2.16
**204**	P 4	79.72	79.72	38.21	90	90	90	38.21	-	2.79
**205**	P 4	78.74	78.74	38.38	90	90	90	15.80	-	2.34
**206**	P 4	78.90	78.90	38.37	90	90	90	55.79	-	2.31
**207**	P 4	78.46	78.46	38.76	90	90	90	38.76	-	1.69
**208**	P 4	78.55	78.55	38.52	90	90	90	38.52	-	1.75
**209**	P 4	78.21	78.21	38.90	90	90	90	26.01	-	1.69
**210**	P 4	78.75	78.75	38.43	90	90	90	38.43	-	2.78
**211**	P 4	78.81	78.81	38.30	90	90	90	78.81	-	1.67
**212**	P 4	78.49	78.49	38.73	90	90	90	55.50	-	2.36
**213**	P 4	78.49	78.49	38.79	90	90	90	31.79	-	1.82
**214**	P 4	78.48	78.48	38.72	90	90	90	27.56	-	2.09
**215**	P 4	78.57	78.57	38.47	90	90	90	38.47	-	2.29
**216**	P 4	78.59	78.59	38.59	90	90	90	21.67	-	2.10
**217**	P 4	78.55	78.55	38.52	90	90	90	38.52	-	2.09
**218**	P 4	78.49	78.49	38.67	90	90	90	25.99	-	1.76
**219**	P 4	78.59	78.59	38.61	90	90	90	34.65	-	2.39

Image #	Space group	a	b	c	α	β	γ	Resolution
**220**	P 4	78.34	78.34	38.39	90	90	90	38.39 - 2.48
**221**	P 4	78.62	78.62	38.48	90	90	90	19.24 - 2.39
**222**	P 4	78.76	78.76	38.40	90	90	90	38.40 - 2.17
**223**	P 4	78.84	78.84	38.24	90	90	90	38.24 - 1.85
**224**	P 4	78.51	78.51	38.74	90	90	90	78.51 - 2.26
**225**	P 4	78.85	78.85	38.41	90	90	90	12.04 - 2.60
**226**	P 4	78.93	78.93	38.28	90	90	90	38.28 - 2.16
**227**	P 4	78.58	78.58	38.61	90	90	90	38.61 - 2.10
**228**	P 4	78.57	78.57	38.58	90	90	90	10.38 - 2.31
**229**	P 4	78.46	78.46	38.45	90	90	90	15.80 - 1.99
**230**	P 4	78.64	78.64	38.86	90	90	90	35.17 - 1.71
**231**	P 4	78.79	78.79	38.36	90	90	90	38.36 - 2.59
**232**	P 4	79.03	79.03	38.36	90	90	90	22.58 - 2.21
